# Bariatric surgery induces pancreatic cell transdifferentiation as indicated by single‐cell transcriptomics in Zucker diabetic rats

**DOI:** 10.1111/1753-0407.13521

**Published:** 2023-12-27

**Authors:** Yongjun Liang, Jason Widjaja, Jiawei Sun, Mengyi Li, Zhengdong Qiao, Ting Cao, Yueqian Wang, Xiong Zhang, Zhongtao Zhang, Yan Gu, Peng Zhang, Jianjun Yang

**Affiliations:** ^1^ Center for Medical Research and Innovation, Shanghai Pudong Hospital Fudan University Pudong Medical Center Shanghai China; ^2^ Fudan Zhangjiang Institute Fudan University Shanghai China; ^3^ Shanghai Key Laboratory of Vascular Lesions Regulation and Remodeling Shanghai China; ^4^ Department of Bariatric and Metabolic Surgery Fudan University Affiliated Huadong Hospital Shanghai China; ^5^ Novogene Bioinformatics Institute Beijing China; ^6^ Division of Metabolic and Bariatric Surgery, General Surgery Center, Beijing Friendship Hospital Capital Medical University Beijing China; ^7^ National Clinical Research Center for Digestive Diseases Beijing China

**Keywords:** bariatric surgery, pancreatic cell differentiation, type 2 diabetes mellitus

## Abstract

**Aims:**

Bariatric surgery results in rapid recovery of glucose control in subjects with type 2 diabetes mellitus. However, the underlying mechanisms are still largely unknown. The present study aims to clarify how bariatric surgery modifies pancreatic cell subgroup differentiation and transformation in the single‐cell RNA level.

**Methods:**

Male, 8‐week‐old Zucker diabetic fatty (ZDF) rats with obesity and diabetes phenotypes were randomized into sleeve gastrectomy (Sleeve, *n* = 9), Roux‐en‐Y gastric bypass (RYGB, *n* = 9), and Sham (*n* = 7) groups. Two weeks after surgery, the pancreas specimen was further analyzed using single‐cell RNA‐sequencing technique.

**Results:**

Two weeks after surgery, compared to the Sham group, the metabolic parameters including fasting plasma glucose, plasma insulin, and oral glucose tolerance test values were dramatically improved after RYGB and Sleeve procedures (*p* < .05) as predicted. In addition, RYGB and Sleeve groups increased the proportion of pancreatic β cells and reduced the ratio of α cells. Two multiple hormone‐expressing cells were identified, the *Gcg+/Ppy +* and *Ins+/Gcg+/Ppy +* cells. The pancreatic *Ins+/Gcg+/Ppy +* cells were defined for the first time, and further investigation indicates similarities with α and β cells, with unique gene expression patterns, which implies that pancreatic cell transdifferentiation occurs following bariatric surgery.

**Conclusions:**

For the first time, using the single‐cell transcriptome map of ZDF rats, we reported a comprehensive characterization of the heterogeneity and differentiation of pancreatic endocrinal cells after bariatric surgery, which may contribute to the underlying mechanisms. Further studies will be needed to elucidate these results.

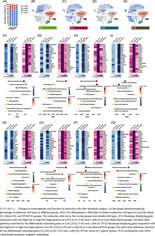

## Graphical abstract

Highlights

- The single‐cell transcriptome map of Zucker diabetic fatty rats' pancreatic endocrine cells after bariatric surgery were reported for the first time.
- An increased ratio of pancreatic β cells, which is associated with other pancreatic endocrine cell types transdifferentiated into β cells, was observed following bariatric surgery.
- For the first time, we identify the elevation of cells in the pancreas following bariatric surgery, which is indirect evidence to support the existence of transdifferentiation.

## 1 INTRODUCTION

Recent studies reported that the worldwide prevalence of diabetes in adults increased from 108 million in 1980 to 422 million in 2014, of which the majority are type 2 diabetes mellitus (T2DM).^1^ T2DM is characterized by hyperglycemia and progressive β‐cell breakdown, and the progress of T2DM is strongly related to obesity.^2^, ^3^ Bariatric surgery has been proven to be the most effective modality to achieve sustained weight loss and remission of obesity‐related diseases including T2DM.^4^, ^5^, ^6^ Currently, sleeve gastrectomy (Sleeve) and Roux‐en‐Y gastric bypass (RYGB) are the most performed bariatric procedures worldwide.^7^

Both Sleeve and RYGB result in the rapid recovery of glucose control and regularly permit discontinuation of antidiabetes medications within days following surgery.^8^ However, although several hypotheses exist regarding the mechanism of diabetes remission following bariatric surgery, we are still far from understanding it.^9^

Previous studies have reported that Sleeve leads to an expansion of the alpha‐cell population that is related to transdifferentiation and improved β‐cell function.^10^, ^11^, ^12^ The sustained T2DM remission relies on the long‐term maintenance of pancreatic islet cells numbers and function. Individual cellular alterations are complex and can be missed when investigated at the level of the entire pancreatic islet.^13^, ^14^ With advancements in technology, single‐cell measurements are able to reveal unsuspected subpopulations or new transcriptional mechanisms. Using the single‐cell RNA‐sequencing technique, we could discover high degrees of gene expression variability within each pancreatic endocrine cells, which have never been tested on bariatric surgery.^13^, ^14^ Due to the difficulties in studying the changes of pancreatic endocrine cells in human bariatric populations, we use Zucker diabetic fatty (ZDF) rats as the experiment subject. The ZDF rat is a genetic animal model for obesity and T2DM and, therefore, deemed suitable for the study of bariatric and metabolic surgery.^15^

## 2 METHODS

### 2.1 Diabetes phenotype identification of ZDF rats and surgical procedures

All applicable animal care and use‐related guidelines were followed, and all procedures were approved by the Institutional Animal Care and Utilization Committee of *our institution*. A total of 33 male, 8‐week‐old, ZDF rats^fa/fa^ and 7 male ZDF rats^fa/+^ (control models) were purchased from Beijing Vital River Laboratory Animal Technology Co. Ltd and housed in a specific pathogen‐free room with standard temperature and humidity. After 1‐week acclimatization and 6 weeks following high‐fat diet, the body weight, fasting blood glucose (FBG), plasma insulin levels, and oral glucose tolerance test (OGTT) excursion of ZDF rats^fa/fa^ and ZDF rats^fa/+^ were measured for phenotyping.

The rats with typical diabetes phenotypes were randomized into Sleeve (*n* = 9), RYGB (*n* = 9), and Sham (*n* = 7) groups. All surgical procedures and postoperative care have been described in a previous study.^16^ In brief, for the RYGB procedure, the small bowel was transected 30 cm from the ligament of Treitz. The distal segment was then anastomosed to the gastric pouch and the proximal segment was anastomosed 10 cm below the gastrointestinal anastomosis. For the Sleeve procedure, the greater curvature along with the fundus was resected until about 0.6 cm away from the pylorus.

### 2.2 Rat pancreas procurement for islet isolation and single‐cell preparation

A midline incision was made on anesthetized rats, and the aortic arch was perfused through ice‐cold preservation solution. The duodenal entrance of the common bile duct was clamped, and the preconfigured collagenase XI (1 mg/mL, C7657, Sigma) was injected into the pancreas until edema. The inflated pancreas was fully detached from the intestine and stomach and immediately placed into a 50 mL tube containing precooled collagenase. The pancreas was bathed in water at 37°C for 15 min, vortexed twice during digestion,n and terminated with complete medium (MEM + 10% FBS + 100 U/mL penicillin +100 μg/mL streptomycin, 11 095 080, 10100139C, 15 140 122, Gibco). Following shaking, centrifugation, and supernatant removal (three times), the suspension was filtered through 400 μm strainer (43–50 400‐50, pluriSelect) into a new 50 mL tube and centrifuged. After removing the supernatant, resuspending with Histopaque 1077 (10 771, Sigma) and adding MEM on the surface of liquid, the density gradient centrifugation was performed, and the islets were obtained from the interface between the two layers. Rat islet samples were fully recovered, enzymatically dissociated into single cells using Accutase (A6964, Sigma), and prepared for subsequent sequencing after cell viability evaluation.

### 2.3 Single‐cell RNA library preparation and sequencing

Single‐cell RNA libraries were constructed according to the standard protocol of Chromium Single Cell 3’ Reagent Kit v2 (PN‐120237, PN‐120236 and PN120262, 10x Genomics) on 10x Chromium Single Cell platform. Briefly, the cell concentration and viability were quantified, and the appropriate number of cells, aiming for targeted cell recovery of 5000–8000 cells per reaction, were loaded into the Chromium Chip. After running the Chromium Controller, the Gel Beadsin‐emulsion (GEMs) were generated and then transferred to emulsion‐safe strip tubes for GEM‐RT using a thermal cycler. Following barcoding, the full‐length cDNAs were cleaned with Dynabeads, amplificated, purified using SPRIselect reagent, and quantified on an Agilent TapeStation. Subsequently, the single‐cell 3′ gene expression library was constructed through the following steps: (a) fragmentation, end repair, and A‐tailing; (b) postfragmentation end repair and A‐tailing double‐sided size selection; (c) adaptor ligation and postligation cleanup; (d) sample index polymerase chain reaction (PCR) and post‐sample index PCR double‐sided size selection; and (e) postlibrary construction quality control. The prepared libraries were sequenced with a NovaSeq 6000 System (Illumina) at the recommended depth.

### 2.4 Single‐cell RNA‐seq data analysis

Single‐cell raw sequencing data was generated into feature‐barcode matrices through Cell Ranger (v6.0, 10X Genomics) pipeline with default parameters. Then R package Seurat (v3.6.3, Satija Lab and Collaborators) was utilized for subsequent analysis. Raw gene expression matrices from the cartridge were read into R and converted to Seurat objects. High‐quality single cells that had unique feature counts between 200 and 6000, with unique molecules >500, and <20% mitochondrial counts were qualified and retained. In the end, 53 373 cells remained for further analysis. The gene expression matrix was then normalized to the total cellular unique molecular identifier count. The top 2000 features were selected as highly variable genes for further clustering analysis. To reduce dimensionality, pPrincipal component analysis was performed based on the highly variable genes after scaling the data with respect to unique molecular identifier counts. Then t‐distributed stochastic neighbor embedding or uniform manifold approximation and projection algorithm was utilized for further reduce dimensionality. The transcriptional markers of each cluster were calculated using the FindAllMarkers function with the Wilcoxon test under the following criteria: log2 fold change >0.25; min. pct >0.25. Feature plots, violin plots, and heatmap were utilized to show clustering results. Average expression of indicated genes in each cluster or cell type was calculated with the AverageExpression function, and the heatmap was drawn with R package heatmap with default parameters.

### 2.5 Pseudotime trajectory with monocle

After cell type annotation, single‐cell pseudotime analysis was performed for each cell type separately using Monocle2 (v2.4.0, Cole Trapnell and Davide Cacchiarelli) with DDR‐Tree reduction method. As for nonimmune cells, single‐cell pseudotime analysis was conducted with default parameters to eliminate batch effect. Briefly, raw gene expression matrix of pancreatic endocrine cells was converted into a Monocle object. During feature selection, differentially expressed genes were chosen between cell types as ordering genes for downstream analysis. Trajectory plots, gene kinetics plots, and heatmap were utilized to show pseudotime results.

### 2.6 SCENIC analysis

SCENIC package (v1.1.2, Laboratory of Computational Biology) was employed to infer gene regulatory networks by transcription factors. The log‐normalized expression matrix of α cells, β cells, and *Ins+/Gcg+/Ppy +* cells (or α cells, γ cells and *Gcg+/Ppy +* cells) was used as input to unveil regulatory information. First, the coexpression networks were calculated and generated using GRNBoost2. Subsequently, the regulons for each transcription factor were further identified by RcisTarget. After scoring the regulon activity for each cell by AUCell, a heatmap was generated for visualization. Finally, the differentially activated regulons in each cell type were identified by Wilcoxon test.

### 2.7 Cell–cell communication analysis

The cell–cell interactions between pancreatic endocrine cell types, including α cells, β cells, δ cells, γ cells, *Gcg+/Ppy +* cells, and *Ins+/Gcg+/Ppy +* cells, were systematically analyzed using a python‐based tool CellPhoneDB (v2.1.7, Teichmann Lab). As a manual curated repository of ligands, receptors, and their interactions for human proteins, the ortholog genes of rat transcriptome dataset were used for ligand‐receptor pairs prediction. The potential interaction strength between two cell types was calculated based on the expression of ligand receptor pairs with the lower cutoff for expression proportion of any ligand or receptor set to 10%. The enriched ligand‐receptor pairs were computed based on permutation test with 1000 times, extracted with *p* value <.01 and visualized with the dot plot. The whole interaction network was generated by Cytoscape (v3.8.2, Agilent and IBS).

### 2.8 Statistical analysis

All statistical analysis was performed using SPSS software (v22.0, SPSS Inc.). The data were expressed as means ± SEM. Comparisons between multiple groups were conducted using one‐way analysis of variance with the Tukey multiple comparison test. Statistical significance was set at *p* < .05.

## 3 RESULTS

### 3.1 Metabolic surgery ameliorates diabetes phenotypes

**FIGURE 1:**
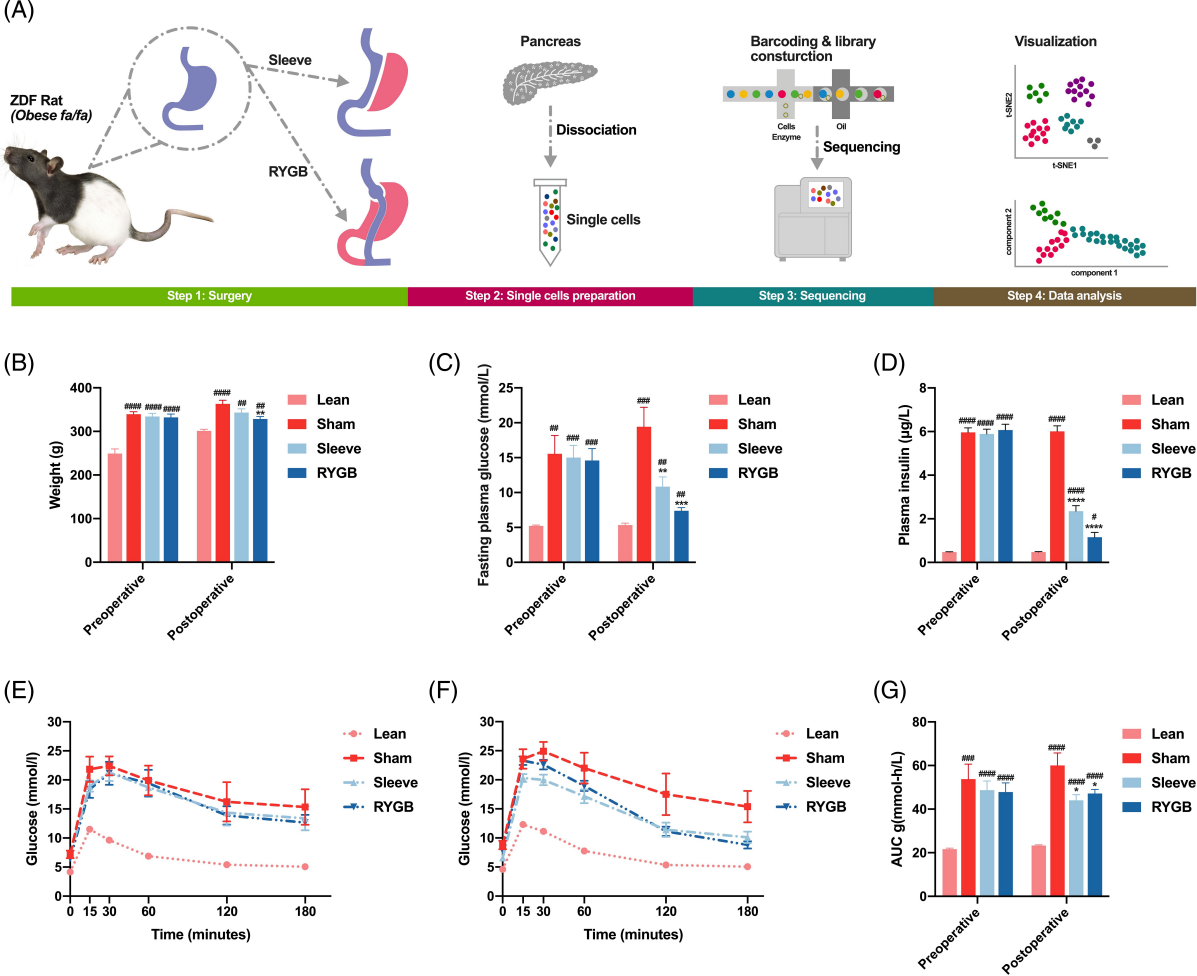
Characterization of diabetes phenotype and surgical outcomes. (A) Schematic diagram of the whole single‐cell RNA‐seq project. (B‐G) Weight (B), FBG (C), plasma insulin (D), OGTT before surgery (E), OGTT after surgery (F), and area under curve (AUC) of OGTT glucose excursion (G). All values were shown as mean ± SEM. * for metabolic surgery groups versus sham group, # for metabolic surgery groups or sham group versus control group (*p < .001). RYGB, Roux‐en‐Y gastric bypass; ZDF, Zucker diabetic fatty.

The whole workflow was shown in Figure 1A. Compared to the ZDF rats^fa/+^ (Lean group), high‐fat diet induction successfully established typical diabetes phenotypes in the ZDF rats^fa/fa^ (Sham, Sleeve, and RYGB group) with elevated body weight, hyperglycemia, and hyperinsulinemia (Figure 1B‐E and Figure 1G). Two weeks after surgery, compared to the Sham group, body weight was significantly decreased only in the RYGB group (*p* < .05) (Figure 1B). The remaining metabolic parameters including FBG, plasma insulin, OGTT, and area under the curve of OGTT glucose excursion were dramatically improved after RYGB and Sleeve procedures (Figure 1C,D,F,G), ensuring the authenticity of subsequent pancreatic single‐cell sequencing.

### 3.2 Metabolic surgery resulted in multiple changes within the pancreatic endocrine cells

**FIGURE 2:**
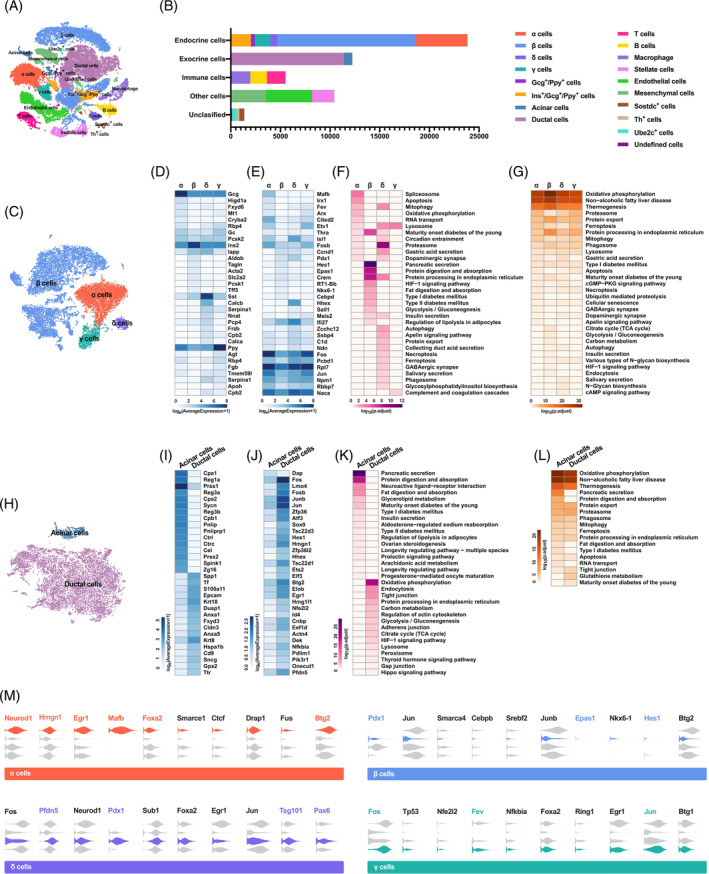
Single‐cell transcriptome analyses of rat pancreas. (A) Two‐dimensional tSNE plot of all cells (*n* = 53 373) based on the expression of highly variable genes. (B) Histogram showing the number of different cell types. (C) Projection of endocrine cells (*n* = 21 409) onto two dimensions using t‐SNE based on the expression values of genes with highest biological variation across cells. (D and E) Heatmaps with expression distributions for enriched genes (D) and TFs (E) in each endocrine cell type. (F and G) Pathways and functional gene sets with enriched genes (F) or abundant genes (G) in α cells, β cells, δ cells, and γ cells. (H) t‐SNE map of exocrine cells including acinar and ductal cells (*n* = 12 235). (I and J) Heatmaps showing expression distributions for enriched genes (I) and TFs (J) in exocrine cell types. (K and L) Heatmaps of the pathways generated with enriched genes (K) or abundant genes (L) in acinar and ductal cells. (M) Violin plots for expression of upstream regulators of enriched genes in each endocrine cell type. TCA, tricarboxylic acid; TF, transcription factor; tSNE, t‐distributed stochastic neighbor embedding.

By analyzing gene expression variation of the all‐filtered cells in an unbiased manner, the single‐cell transcriptome data was visualized with t‐distributed stochastic neighbor embedding (t‐SNE) (Figure 2A,B). To present traditional pancreatic endocrine cells clearly, a t‐SNE map embodying α cells, β cells, δ cells, and γ cells was generated by reanalyzing the gene expression variation of 21 406 cells (Figure 2C). Apparently, the transcriptomes of the four types of endocrine cells are significantly distinct. For β cells, there is a strong internal heterogeneity with several subclusters (Figure 2C). By comparing the transcriptome differences between a specific cell type and the remaining endocrine cell types, enriched genes were screened out, and transcription factors (TFs) were further identified using the TFcheckpoint database (Figure 2D,E). Defined endocrine cell markers GCG, INS, SST, and PPY were enriched in α cells, β cells, δ cells, and γ cells, respectively. In addition, seven other cell‐specific genes for every endocrine cell type were shown in Figure 2D. Compared with the other cell types, δ cells had the largest number of enriched genes; however, most of their functions were unclear.

TFs are vital for determining cell fate, characteristics, and functions by orchestrating intracellular gene expression programs. Thus, the cell type specificities of TFs were investigated by running the same pipeline as enriched genes. In terms of quantity, α and δ cells had more enriched TFs, about 50 each, whereas β and γ cells had only 12 each. A total of 32 cell‐specific TFs were listed and visualized with a heatmap, including 9 in α cells, 8 in β cells, 8 in δ cells, and 7 in γ cells (Figure 2E). In general, α and γ cells had a more similar TFs expression patterns, while δ cells were the most unique (Figure 2E). CITED2 and THRA were two newly identified TFs restricted to the α cells. For β cells, except for two well‐known cell‐specific TFs PDX1 and NKX6‐1, CCND1, HES1, EPAS1, CREM, RT1‐BB, and CEBPD were detected. Whereas for the δ cells, several novel signature TFs such as SALL1, IFI27, ZCCHC12 etc. were enriched. Moreover, the TF analysis demonstrated that although some TFs had slightly higher expression levels in γ cells, the specificity was low. These TFs, including FOS, PCBD1, RP17, JUN, NPM1, RBBP7, and NACA, were more like panendocrine markers. This single‐cell transcriptome dataset not only recapitulated the known cell‐type specific genes and TFs but also discovered novel ones deserving further investigation for their roles in cellular identity and function.

To further clarify the correlation between cell‐specific genes and the function execution of endocrine cells, ingenuity pathway analysis (IPA) was conducted. Consistent with the two core functions of β cells, including glucose sensing and insulin secretion, enriched genes were involved in pathways of insulin secretion, glycolysis/gluconeogenesis, pancreatic secretion, T2DM, etc., contributing to metabolic homeostasis (Figure 2F). IPA of α cell‐specific genes demonstrated that the pathways related to cell proliferation, apoptosis, metabolism, and secretion were enriched (Figure 2F). Because δ cells had the most enriched genes, the pathways associated also covered the most functions. Some of them overlapped with α cells related to diabetes, and the rest were specific to δ cells (Figure 2F).

In addition to the enriched genes, the transcripts with the highest expression abundance in every endocrine cell type were identified, and the pathways they were involved in were also analyzed. In theory, these abundantly expressed genes were more inclined to participate in cell‐based survival and metabolic functions, such as oxidative phosphorylation, protein processing, apoptosis, carbon metabolism, etc., which were confirmed in all endocrine cell types (Figure 2G).

As endocrine and exocrine organs, the function of acinar and duct cells is also crucial for the pancreas to maintain metabolic homeostasis. By calculating the cellular transcriptome differences, 12 235 cells were naturally divided into acinar and ductal cells and projected onto two dimensions using t‐SNE (Figure 2H). Following the same analysis pipeline for endocrine cells, the cell‐specific genes were detected and visualized with a heatmap (Figure 2I). On the list, other than *Cpa1, Reg1a, Prss1, Reg3a, Cpa2, Pnlip, and Spink1*, the rest were brand new signature genes meriting further verification and investigation (Figure 2I). For ductal cells, 4 out of 16 including *Spp1, Krt18, Krt8, and Cd9*, were known genes with specificity, had robust expression in ductal cells (Figure 2I). There were hundreds of enriched TFs in ductal cells, such as ID4, HHEX, SOX9 etc. but almost none in acinar cells (Figure 2J). The complex network of TFs maintains the high plasticity of ductal cells, which may contribute to acinar‐to‐ductal metaplasia and ductal cell reprogramming to insulin‐producing β‐like cells.

IPA analysis revealed that the enriched genes of acinar cells contributed to pathways relevant to digestive enzyme secretion or metabolism, such as pancreatic secretion, protein digestion and absorption, fat digestion and absorption, insulin secretion, type 2 diabetes, etc. (Figure 2K). For ductal cells, pathways of oxidative phosphorylation, endocytosis, tight junction, adherent junction, gap junction, etc. were annotated and responsible for collecting and delivering digestive enzymes (Figure 2K). Moreover, except for pancreatic secretion and protein digestion and absorption, the pathways that abundantly expressed genes of acinar and ductal cells involved in were similar, mainly referring to metabolic functions (Figure 2K).

To further understand the specificity of pancreatic endocrine cell function in regulating the metabolic homeostasis, upstream regulators of enriched genes were investigated using IPA analysis. Numerous upstream regulators with different molecule types were detected, and then TFs were extracted and compared with the single‐cell transcriptome dataset. In each type of endocrine cells, 10 TFs, upstream regulators determining cell identity, were listed and their expressions displayed with violin plots (Figure 2M). Those TFs that were both enriched genes and upstream regulators were highlighted by colors.

**FIGURE 3:**
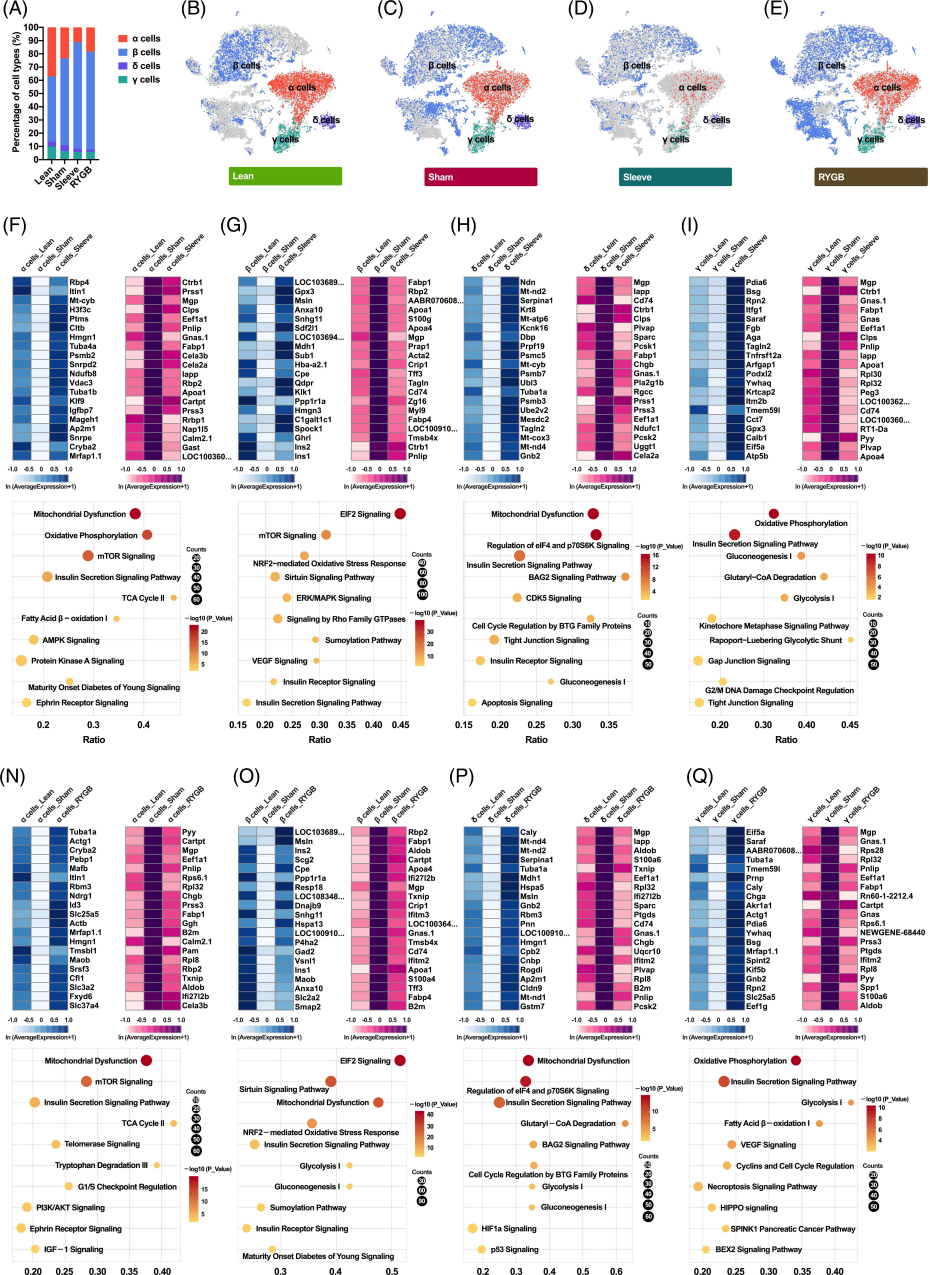
Changes in transcriptome and function of endocrine cells after metabolic surgery. (A) Bar graph demonstrating the percentage of endocrine cell types in different groups. (B‐E) Two‐dimensional t‐SNE plots of distinct endocrine cell types in Lean (B), Sham (C), Sleeve (D), and RYGB (E) groups. The endocrine cells not in the current group were shaded with gray. (F‐I) Heatmaps displaying gene expression with low‐high‐low or high‐low‐high patterns of α (F), β (G), δ (H) and γ cells (I) in Lean‐Sham‐Sleeve groups. Dot plots show pathways enriched by the differentially expressed genes in α (F), β (G), δ (H) and γ cells (I). (N‐Q) Heatmaps displaying gene expression with low‐high‐low or high‐low‐high patterns of α (N), β (O), δ (P) and γ cells (Q) in Lean‐Sham‐RYGB groups. Dot plots show pathways enriched by the differentially expressed genes in α (N), β (O), δ (P) and γ cells (Q). RYGB, Roux‐en‐Y gastric bypass; TCA, tricarboxylic acid; tSNE, t‐distributed stochastic neighbor embedding.

Recovery of pancreatic islet function, especially the regeneration and reprogramming of β cells is observed following bariatric surgery. To figure out the functional shift, the single‐cell transcriptomes of all endocrine cell types were investigated by comparison between groups. Compared with the Sham group, RYGB and Sleeve groups increased the proportion of β cells and reduced the ratio of α cells (Figure 3A). In addition to the quantitative changes, the characteristics of endocrine cells also changed after surgery, especially β cells (Figure 3B–E).

To figure out the possible internal cause, the investigation of single‐cell transcriptomes of pancreatic endocrine cells was conducted and the genes showed different expression patterns (each group was screened and displayed with heatmaps) (Figure 3F–I and Figure 3N–Q). Overall, the transcriptome variations caused by the two surgeries were partly similar (data not shown). Four upregulated genes (*Cryba2*, *Itln1*, *Mrfap1.1*, and *Hmgn1*) and nine downregulated genes (*Cartpt*, *Mgp*, *Eef1a1*, *Pnlip*, *Prss3*, *Fabp1*, *Calm2.1*, *Rbp2*, and *Cela3b*) were both detected in pancreatic cells after Sleeve and RYGB surgery (Figure 3F,N).

For pancreatic β cells, eight upregulated genes (*LOC103689940*, *Msln*, *Ins2*, *Cpe*, *Ppp1r1a*, *Snhg11*, *Ins1*, and *Anxa10*) and 10 downregulated genes (*Rbp2*, *Fabp1*, *Apoa4*, *Mgp*, *Crip1*, *Tmsb4x*, *Cd74*, *Apoa1*, *Tff3*, and *Fabp4*) were both detected after Sleeve and RYGB surgery (Figure 3G and Figure 3O). In summary, metabolic surgery resulted in multiple changes within the pancreatic endocrine cells, which could be related to the control of blood glucose homeostasis.

### 3.3 Subpopulation identification and distribution under different metabolic stress

**FIGURE 4:**
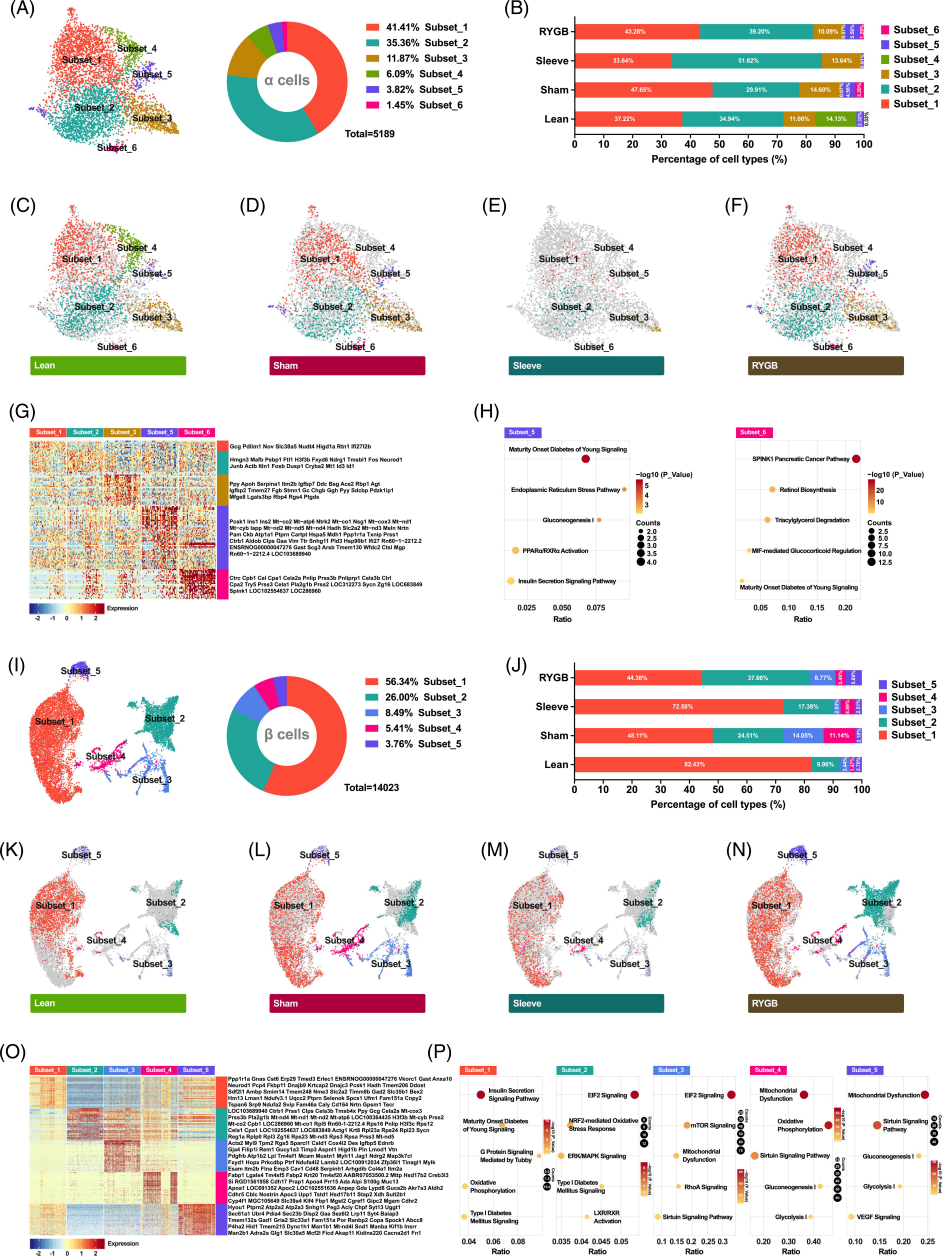
Uncovering and characterizing subpopulation of α and β cells. (A) Two‐dimensional t‐SNE representing six subsets of α cells (*n* = 5189) based on the expression of highly variable genes (left). Doughnut chart showing the proportions of different subsets (right). (B) Bar graphs showing the percentage of different subsets of α cells in different groups. (C‐F) Two‐dimensional t‐SNE plots of distinct subsets of α cells in Lean (C), Sham (D), Sleeve (E), and RYGB (F) groups. (G) Heatmap showing subpopulation‐specific genes of different α cell subsets. (H) Dot plots showing pathways enriched by the subpopulation‐specific genes of subset_5 and subset_6. (I) Two‐dimensional t‐SNE representation of five subsets in β cells (*n* = 14 023) (left). Doughnut chart showing the proportions of different subsets (right). (J) Bar graphs showing the percentage of different subsets of β cells in different groups. (K‐N) t‐SNE maps of distinct subsets of β cells in Lean (K), Sham (L), Sleeve (M), and RYGB (N) groups. (O) Heatmap showing enriched genes of different β cell subsets. (P) Dot plots show pathways annotated by the enriched genes of different subsets in β cells. RYGB, Roux‐en‐Y gastric bypass; tSNE, t‐distributed stochastic neighbor embedding.

Theoretically, each cell is a unique unit with a specific transcriptome. Thus, there are often cell subpopulations existing in specific cell types. To explore heterogeneity within cell types, clustering analysis was performed, and robust separation was observed in α and β cells but not in δ and γ cells. A total of 5189 α cells were spontaneously separated into six cell subsets, among which subsets 1, 2, and 3 accounted for nearly 90% (Figure 4A).

Compared with the lean group, both T2DM progression (sham group) and surgical intervention (Sleeve and RYGB groups) caused changes in the proportion of α cell subpopulations (Figure 4B). It was further confirmed by the t‐SNE maps of cell subsets distribution of each group (Figure 4C–F).

To better understand the functional differences of these cell subsets and the causes of cell distribution changes under different metabolic stress, cell‐specific genes were analyzed (Figure 4G). *Subset_1*, which accounted for the highest proportion of total α cells, had the highest *Gcg* expression level (Figure 4A and Figure 4G). Furthermore, the proportion of the *subset_1* was reduced after metabolic surgery (Figure 4B). The *subset_2* was enriched with the most α cell specific genes, such as *Hmgn3*, *Mafb*, *Fxyd6*, *Cryba2*, *Mt1*, etc. (Figure 4G). Unexpectedly, *subset_3* behaved stably under any circumstances, whereas the *subset_4* mainly appeared in the lean group (Figure 4B). *Subset_5* had the strongest heterogeneity with most cell‐specific genes, of which β cell signature genes including *Pcsk1*, *Ins*, *Lapp*, *Slc2a2*, *Aldob*, and *Txnip* were enriched (Figure 4G). Lastly, *subset_6* has the lowest ratio of enriched acinar cell specific genes (*Ctrc*, *Cpb1*, *Cel*, *Cpa1*, *Pnlip*, *Pnliprp1*, *Ctrl*, *Cpa2*, *Prss2*, *Sycn*, *Zg16*, *Spink1*) (Figure 4G). IPA analysis for enriched genes of *subset_5* and *subset_6* was carried out and metabolic pathways were annotated (Figure 4H).

The same analysis process was also performed for β cells. A total of 14 023 cells generated the t‐SNE map with five subpopulations embodied (Figure 4I). Apparently, compared with α cells, β cells were more heterogeneous with more robust and independent subsets displayed. The *subset_1* and *subset_2* accounted for 82.34% of β cells (Figure 4I). Furthermore, T2DM significantly reduced the overall proportion of *subset_1* and *subset_2*, which was elevated following bariatric surgery (Figure 4J). Conversely, the proportions of *subset_3* and *subset_4* were both increased during T2D and decreased after bariatric surgery (Figure 4J). The changes in the distribution of β cell subsets under different metabolic stress were concretely visualized with t‐SNE maps (Figure 4K–N). To elucidate the internal mechanism of the above phenomenon, the cell‐specific genes of every subpopulation were identified. In terms of the number of enriched genes alone, the heterogeneity of *subset_3* (607), *subset_4* (397), and *subset_5* (215) was stronger than *subset_1* (91) and *subset_2* (139). The top 50 were listed in Figure 4O. Pathway analysis revealed that the enriched genes of β cell subpopulations were associated with diabetes and glucose homeostasis (Figure 4P).

### 3.4 Characterization of pancreatic *Gcg*+/*Ppy* + cells and functional change under surgical intervention

**FIGURE 5:**
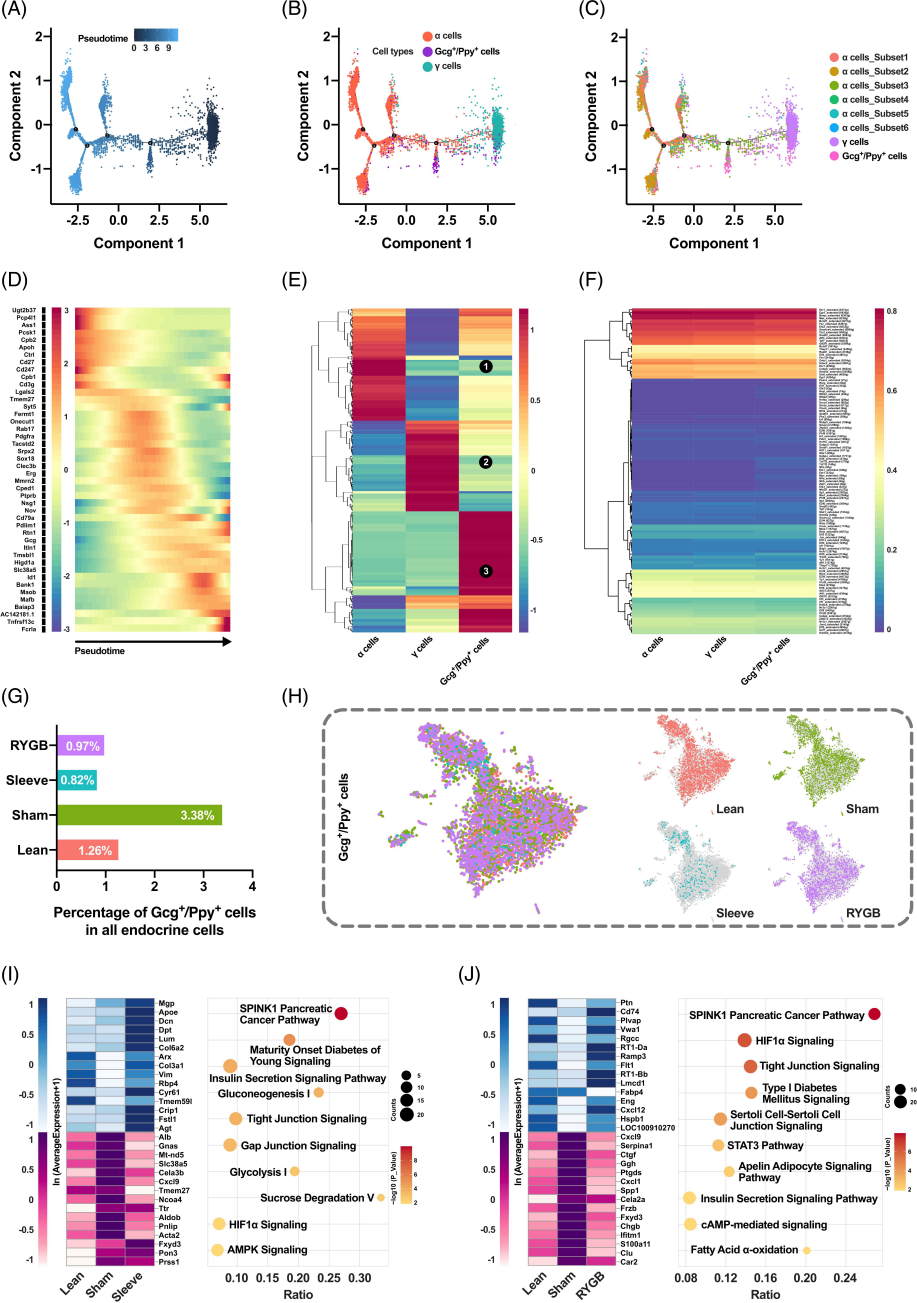
Feature depiction and functional survey of *Gcg*+/*Ppy* + cells. (A–C) Monocle‐generated plots presenting pseudotime ordering (A), trajectory of α, γ, and *Gcg*+/*Ppy* + cells (B), and trajectory embodying α cell subsets (C). (D) Heatmap of genes with branch‐specific, pseudotime‐dependent expression. (E) Clustered heatmap showing the average expression distributions of all genes in α, γ, and *Gcg*+/*Ppy* + cells. (F) Heatmap showing the average expression of TFs inferred by SCENIC in α, γ, and *Gcg*+/*Ppy* + cells. (G) Histogram showing the percentage of *Gcg*+/*Ppy* + cells in all endocrine cells in different groups. (H) t‐SNE maps of *Gcg*+/*Ppy* + cells in different groups. (I‐J) Heatmaps displaying altered gene expression of *Gcg*+/*Ppy* + cells in Lean‐Sham‐Sleeve groups (I) or Lean‐Sham‐RYGB (J) groups. Dot plots show pathways enriched by the differentially expressed genes of *Gcg*+/*Ppy* + cells in Sleeve (I) and RYGB (J) groups. RYGB, Roux‐en‐Y gastric bypass; tSNE, t‐distributed stochastic neighbor embedding.

In addition to the traditional endocrine cells, two multiple hormones‐expressing cells were identified, the *Gcg+/Ppy +* and *Ins+/Gcg+/Ppy +* cells. To further characterize the double positive cells and figure out the relationship between α, γ, and *Gcg+/Ppy +* cells, pseudotime analysis was performed. By ordering the single‐cell transcriptomes of α, γ, and *Gcg+/Ppy +* cells, computational algorithms inferred a pseudotemporal continuum with direction from right to left and four branches were detected (Figure 5A). Mapping the cell types onto the trajectory found that γ cells gathered at the initial point, α cells formed the terminal branches, whereas the *Gcg+/Ppy +* cells were located between the two (Figure 5B). Considering the heterogeneity of α cells, another pseudotime trajectory containing subsets was generated to further clarify the relationship between *Gcg+/Ppy +* cells and α cell subpopulations (Figure 5C). The distribution of *Gcg+/Ppy +* cells and *subset_3* on the pseudotime trajectory was more overlapping, which meant that the transdifferentiation trajectory may be γ cells‐*Gcg+/Ppy +* cells‐*subset_3*‐other subsets of α cells. In the meantime, the pseudotime‐dependent genes were identified and 44 of them were used to generate a heatmap (Figure 5D). The gene expression changed dynamically along with trajectory, such as continuous decline of γ cells enriched genes *Apoh* and *Cpb2* and continued increase of α cells enriched genes *Gcg* and *Mafb*. In addition, the whole transcriptomes of α, γ, and *Gcg+/Ppy +* cells were compared and clustered, which indicated that the *Gcg+/Ppy +* cells had the traits of either α or γ cells as well as their own characteristics (Figure 5E no. 3, denoted by circular black background number three). Dynamic changes of TFs were critical for maintaining cell characteristics or transdifferentiation, which was investigated and displayed with the heatmap (Figure 5F).

To explore the function of *Gcg+/Ppy +* cells under different metabolic stress, its distribution and transcriptome variation were investigated. The proportion of *Gcg+/Ppy +* cells in all pancreatic endocrine cells increased during T2DM but decreased significantly after bariatric surgery (Figure 5G). Similar t‐SNE maps of the double positive cells in different groups suggested few transcriptomes variation and functional difference, which was further confirmed by differentially expressed gene screening (Figure 5H–J). The functions of these few differential genes after surgery were mainly associated with insulin secretion and glucose metabolism (Figure 5I,J).

### 3.5 Characterization and functional survey of pancreatic *Ins+/Gcg+/Ppy +* cells

**FIGURE 6:**
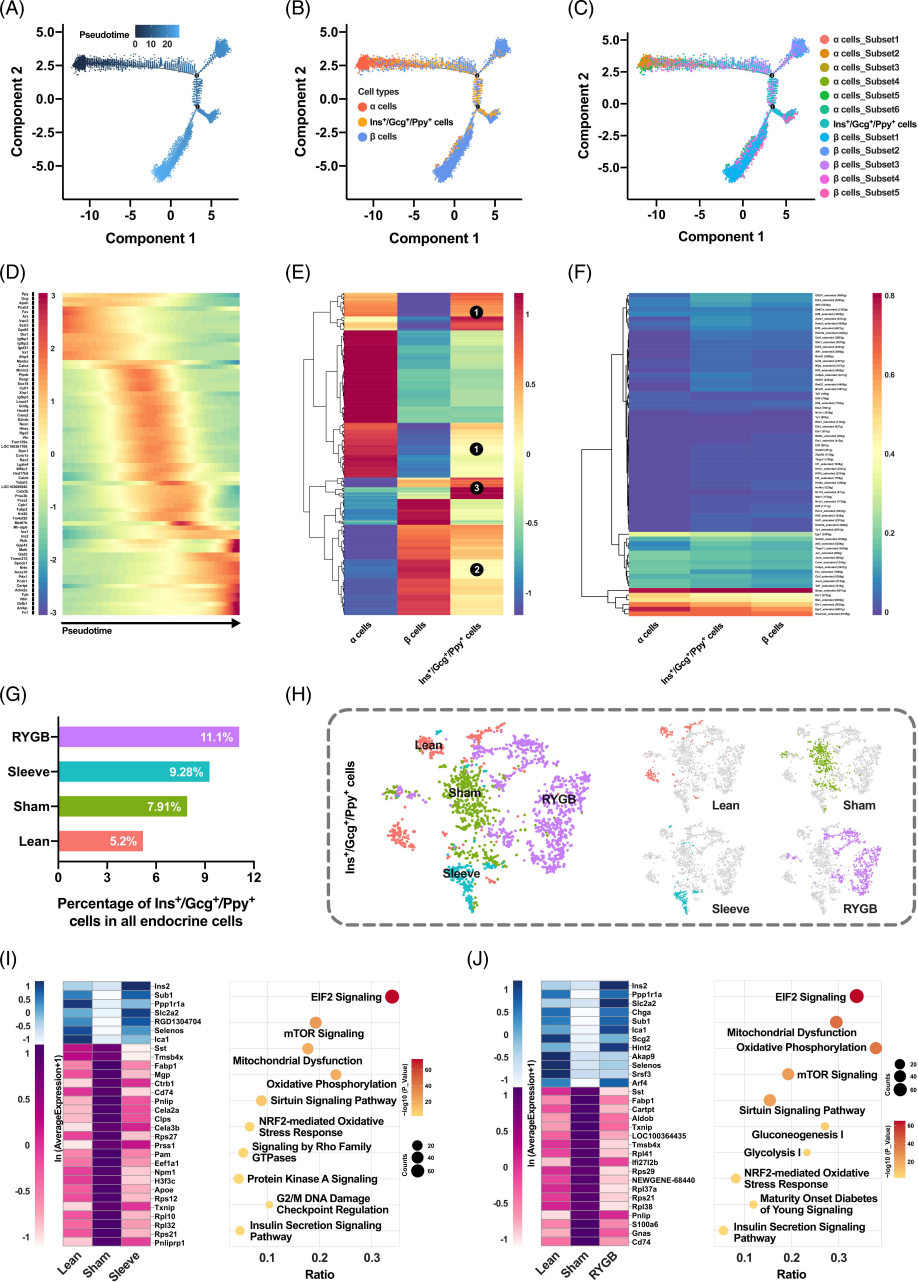
Feature depiction and functional survey of Ins+/*Gcg*+/*Ppy* + cells. (A–C) Monocle‐generated plots presenting pseudotime ordering (A), trajectory (B), and trajectory embodying cell subsets (C). (D) Heatmap of genes with branch‐specific, pseudotime‐dependent expression. (E) Heatmap showing the average expression distributions of all genes in α, β and Ins+/*Gcg*+/*Ppy* + cells. (F) Heatmap showing the average expression of TFs inferred by SCENIC in α, β, and Ins+/*Gcg*+/*Ppy* + cells. (G) Histogram showing the percentage of Ins+/*Gcg*+/*Ppy* + cells in all endocrine cells in different groups. (H) Two‐dimensional t‐SNE representation of Ins+/*Gcg*+/*Ppy* + cells in different groups. (I‐J) Heatmaps displaying gene expression with low‐high‐low or high‐low‐high patterns of Ins+/*Gcg*+/*Ppy* + cells in Lean‐Sham‐Sleeve groups (I) or Lean‐Sham‐RYGB (J) groups. Dot plots present pathways annotated by the differentially expressed genes of Ins+/*Gcg*+/*Ppy* + cells in Sleeve (I) and RYGB (J) groups. RYGB, Roux‐en‐Y gastric bypass; TF, transcription factor; tSNE, t‐distributed stochastic neighbor embedding.

In this study, the pancreatic *Ins+/Gcg+/Ppy +* cells were defined for the first time in single‐cell transcriptome study. The pseudotemporal continuum was depicted for α, β and *Ins+/Gcg+/Ppy +* cells with direction from α to β cells and the triple‐positive cells was located in the middle of the trajectory (Figure 6A,B). To further characterize the *Ins+/Gcg+/Ppy +* cells and figure out its role in heterogeneity of α and β cells, the subpopulations of α and β cells were mapped to the trajectory (Figure 6C). The *Ins+/Gcg+/Ppy +* cells overlapped the most with *subset_5* of α cells. In contrast, the distribution of the triple‐positive cells highly tended to *subset_3* and *subset_4* of β cells. The heatmap showed the gradual expression changes of pseudotime‐dependent genes, especially the reverse changes of α (*Gcg, Pcsk2, Fev, Arx, Irx1*) and β (*Ins, Pdx1, Pck1*) enriched genes (Figure 6D). Moreover, investigation of the whole transcriptomes indicated that the triple‐positive cells not only had similarities with α (Figure 6E no. 1, denoted by circular black background number one) or β (Figure 6E no. 2) cells but also had unique gene expression patterns (Figure 6E *no. 3*).

To gain insight into the molecular regulation of transdifferentiation, the TF expression patterns of the three cell types were investigated (Figure 6F). Interestingly, the percentage of triple‐positive cells was elevated during T2DM and was elevated even higher after bariatric surgery (Figure 6G). The t‐SNE maps manifested huge transcriptome variations, which meant that the functions of *Ins+/Gcg+/Ppy +* cells changed under different metabolic stress (Figure 6H). Lastly, differentially expressed genes and enriched pathways were identified. The insulin secretion and glucose metabolism related genes and pathways were restored after Sleeve and RYGB (Figure 6I,J).

### 3.6 Dynamic reciprocal interaction between pancreatic endocrine cells

**FIGURE 7:**
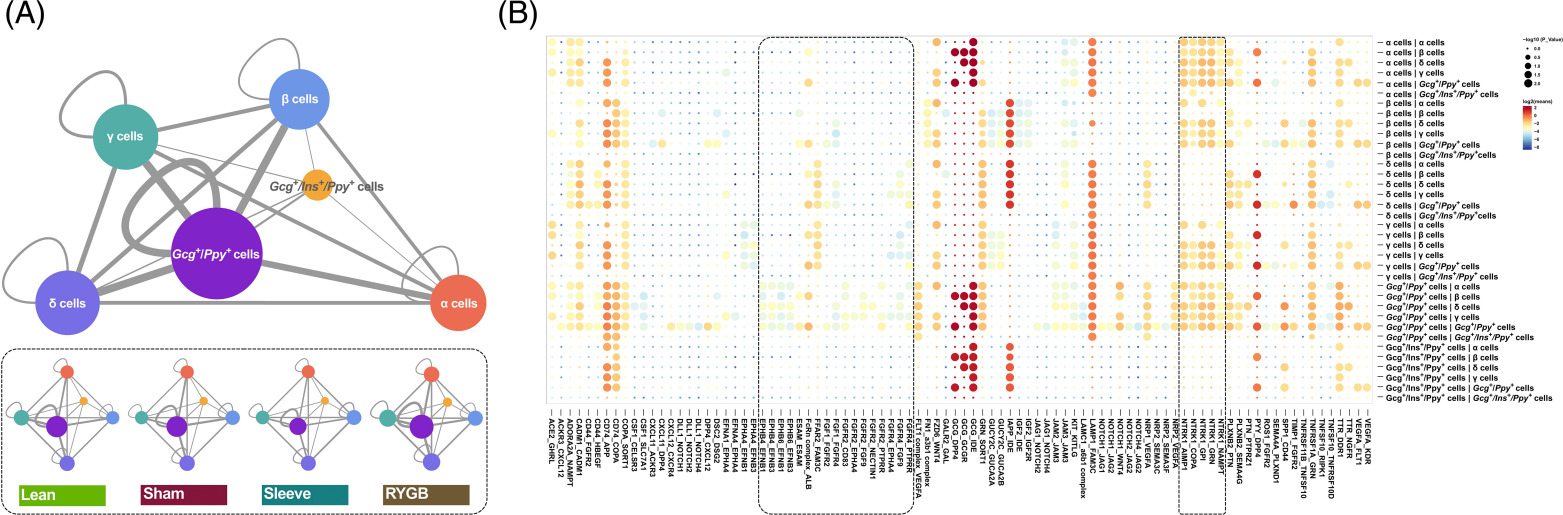
Dynamic interaction between pancreatic endocrine cells. (A) Interaction network of pancreatic endocrine cells analyzed by CellPhoneDB and projected with Cytoscape. (B) Overview of statistically significant interaction pairs between pancreatic endocrine cell types. Size indicated *p* values (permutation test), and color indicated the means of the receptor‐ligand pairs between two cell types. RYGB, Roux‐en‐Y gastric bypass.

To reveal the intercellular dynamic interaction, the potential ligand‐receptor pairs in different cell types were predicted and the cell–cell communications were visualized (Figure 7A). A total of 634 ligand‐receptor pairs were included in this network and the *Gcg+/Ppy +* cells showed the greatest degree in the network. The four types of traditional pancreatic endocrine cells had similar numbers of interaction pairs (~200), whereas the *Ins+/Gcg+/Ppy +* cells had only 38 pairs. Furthermore, the endocrine cell communication of each group was analyzed, and the results showed that they had similar patterns under different metabolic stresses including diabetes or surgical intervention, but the number of interacting pairs was slightly different, with the RYGB group having the most (Figure 7A).

To further clarify the interaction pattern in different cell–cell pairs, a dot plot embodied the entire ligand‐receptor pairs was generated (Figure 7B). Consistent with the interaction network, the communication patterns of the *Gcg+/Ppy +* cells were the most complicated, and conversely, the *Ins+/Gcg+/Ppy +* cells were the simplest. For β cells, its functions were finely regulated by α cells, *Gcg+/Ppy +* cells, and *Ins+/Gcg+/Ppy +* cells through *GCGIDE*, *GCG‐GCGR*, and *GCG‐DPP4*, and also by δ cells, γ cells, *Gcg+/Ppy +* cells, and I*ns+/Gcg+/Ppy +* cells through *PYY‐DPP4*. Similarly, α cells received multiplex signals from β cells (TTR‐DDR1, IAPP‐IDE, CD74‐APP, etc.), δ cells (LAMP1‐FAM3C, IAPP‐IDE, FFAR2‐FAM3C, etc.), *Gcg+/Ppy +* cells (TTR‐DDR1, LAMP1‐FAM3C, GCG‐IDE, CD74‐APP, etc.), and *Ins+/Gcg+/Ppy +* cells (TTR‐DDR1, IAPP‐IDE, GCG‐IDE, etc.). It was worth noting that the ligand‐receptor pairs within the dashed box had the largest variation between different groups, which suggest that these interactions were critical for the information exchange and functional adjustments of endocrine cells when dealing with different metabolic pressures.

## 4 DISCUSSION

Salvage and preservation of the numbers of pancreatic β cell are strongly related to the efficacy and durability of T2DM remission induced by metabolic and bariatric surgery. Pancreatic single‐cell transcriptomics is a promising technology to identify how the surgery alters pancreatic endocrine cell transdifferentiation and regeneration. From our own perspectives, the present study is the first to explore pancreatic single‐cell transcriptomics following the two most commonly performed bariatric procedures, sleeve gastrectomy and RYGB. As suggested in previous study, there are multiple routes in restoring functional β cell mass, which includes transdifferentiation of other cell types into insulin‐producing β‐like cells.^17^ Considering the close correlation between α and β cells, α cell trans‐differentiation has drawn much attention as the source for β cell regeneration.^18^ In the current study, when compared to Sham, the RYGB and Sleeve groups increased their proportion of β cells and reduced their ratio of α cells (Figure 3A). Furthermore, the characteristics of the β cells also altered after surgery (Figure 3B‐E), suggesting that the heterogeneity of β cells became more obvious under T2DM or surgical intervention. We also found 10 upstream regulators of enriched genes in each endocrine cell group, namely *Neurod1* in α cells, Pdx‐1 and *Nkx6.1* in β cells, and Pdx‐1 in δ cells. PDX‐1, Nkx6.1, and MAFA are β‐cell specific TFs that can promote α cells and δ cells to transdifferentiate into β cells.^19^, ^20^, ^21^ Although controversy still exists, upsurge in PDX1 might resulted in the β‐cell mass expansion through their roles in cell proliferation and function as well as with the increased release of glucagon‐like peptide‐1.^12^, ^22^

Thus, bariatric surgery might result in improved glucose control through upregulation of β‐cell TFs and promoting transdifferentiation from α cells and δ cells into β cells. Further research can help to identify the significance of these changes in relation to bariatric surgery.

Surprisingly, in the current study, we did not find *Gcg+/Ins +* cells, which could be further evidence of transdifferentiation from α to β cells,^19^ but *Ins+/Gcg+/Ppy +* cells. Pancreatic α cells are extremely plastic cells, which can convert directly into β cells through epigenetic reprogramming when β cells are impaired.^23^, ^24^, ^25^ In the current experiment, the use of ZDF rats in this study perhaps did not result in pancreas deterioration to the extent of complete β cells ablation,^26^ which could be the reason the *Gcg+/Ins +* cell cells were not found.

In the current study, we found *Gcg+/Ppy +* cells (component of γ cells) that were increased during T2DM; however, they decreased significantly after bariatric surgery (Figure 5G). A study in mice reported that γ ‐cells show a functional cell plasticity similar to α and δ cells.^27^ Intriguingly, it has also been reported that following β cell injury induction, γ cells demonstrated gene expression alterations that engaged in insulin production.^27^ We acknowledged that our current study is not feasible to confirm the possible transdifferentiation of γ cells into insulin‐producing β cell, either directly or indirectly. Further studies are needed to determine the significance of the changes observed in our current study.

Our study accounts the pancreatic *Ins+/Gcg+/Ppy +* cells for the first time through single‐cell transcriptome study. The *Ins+/Gcg+/Ppy +* cells overlapped the most with *subset_5* of α cells, a subpopulation with partial β cell characteristics and a potential for α to β cell transdifferentiation. On the other hand, the distribution of the triple‐positive cells highly tended to *subset_3* and *subset_4* of β cells, which had stronger plasticity in transdifferentiation, dedifferentiation, redifferentiation, and endothelial mesenchymal transition. We also found that the percentage of triple‐positive cells was raised during T2DM and even higher after bariatric surgery (Figure 6G). We speculate that the former might be due to the compensatory effect of insufficient insulin secretion and the latter may be for better β‐cell regeneration, further study will be needed to confirm. Furthermore, the heatmaps displaying the gene expression with low‐high‐low or high‐low‐high patterns of *Ins+/Gcg+/Ppy +* cells and the dot plots presenting pathways annotated by the differentially expressed genes of *Ins+/Gcg+/Ppy +* cells in bariatric surgery groups (Figure 6I,J) could suggest that the *Ins+/Gcg+/Ppy +* cells participated in the recovery of metabolic homeostasis through improvements in the distribution and function after surgery.

Six cell subsets were introduced in α cells, with subsets 1, 2, and 3 accounting for nearly 90%. The subsets 4, 5, and 6 were more separated from the main body of the cell population on t‐SNE map, which suggests that they were relatively more heterogeneous (Figure 4A). *Subset_1* of α cells accounted for the highest proportion of total α cells and had the highest *Gcg* expression level, was increased in the Sham group and reduced following bariatric surgery (Figure 4A, G). Therefore, *subset_1* of α cells might play a crucial role in T2DM progression and alleviation following surgery. Most interestingly, *subset_5* of α cells had β‐cell signature genes that were enriched (Figure 4G). This suggested that these α cells could gain partial β‐cell characteristics and have the potential for α‐ to β‐cell transdifferentiation, which was confirmed by subsequent pseudotime analysis (Figure 6C). *Subset_6* of α cells has the lowest ratio of enriched acinar cell specific genes, which suggests that there could be transdifferentiation between α and acinar cells. This phenomenon has never been reported so far and merits further study.

Five cell subsets were introduced in β cells, with *subset_1* and *subset_2* accounting for 82% (Figure 4I). T2DM significantly reduced the overall proportion of *subset_1* and *subset_2* of β cells, which was elevated following bariatric surgery (Figure 4J). Thus, the two subsets of β cells could be responsible for the impaired insulin secretion during T2DM progression and restored glucose homeostasis after bariatric surgery. The proportions of *subset_3* and *subset_4* of β cells were much lower when compared with *subset_1* and *subset_2*, was increased during T2DM and decreased after bariatric surgery, which suggested that these two subsets may be compensatory β cells. In general, under different metabolic stress, the α and β cell subpopulations will undergo adaptive changes in the cell distribution. Furthermore, bariatric surgery robustly restored the disordered proportions of α and β cell subsets observed during T2DM.

Our current study is mostly limited by its descriptive nature. The current study attempts to investigate the prospect of pancreatic endocrine cells differentiation shortly following bariatric surgery. However, further studies are needed to confirm the relation between the alteration observed in relation to the diabetes remission outcome observed following bariatric surgery. Moreover, we used ZDF rats in this study, as we believed ZDF rats can mimic the obesity and T2DM conditions observed in humans.

## 5 CONCLUSION

By using the single‐cell transcriptome map of ZDF rats, we reported a comprehensive characterization of the heterogeneity and differentiation of pancreatic endocrine cells after bariatric surgery. Bariatric surgery can cause an increase of the ratio of pancreatic β cells and improvement of pancreatic function, which is associated with other pancreatic endocrine cell type transdifferentiate into β cells. For the first time, we identify the elevation of cells in the pancreas following bariatric surgery, which is indirect evidence to support the existence of transdifferentiation. Further research will still be needed to validate the underlying mechanisms and evidence in the level of histopathology.

## AUTHOR CONTRIBUTIONS

Yongjun Liang: Conceptualization, Methodology, Validation, Formal analysis, Investigation, and Writing ‐ Original Draft. Jason Widjaja: Conceptualization, Validation, Formal analysis, Investigation, and Writing ‐ Original Draft. Jiawei Sun: Validation, Formal analysis, Investigation, and Resources. Mengyi Li: Investigation, Resources, Data Curation, and Writing ‐ Review & Editing. Zhengdong Qiao: Formal analysis, Investigation, Data Curation, and Writing ‐ Review & Editing. Ting Cao: Formal analysis, Investigation, and Data Curation. Yueqian Wang: Formal analysis, Investigation, and Data Curation. Xiong Zhang: Formal analysis, Investigation, and Data Curation. Zhongtao Zhang: Conceptualization, Methodology, Validation, and Supervision. Yan Gu: Conceptualization, Methodology, Validation, and Supervision. Peng Zhang: Conceptualization, Methodology, Validation, Writing ‐ Review & Editing, and Supervision. Jianjun Yang: Conceptualization, Methodology, Validation, Writing ‐ Review & Editing, Supervision, and Funding acquisition.

## FUNDING INFORMATION

This research is funded by Fudan Zhangjiang Clinical Medicine Innovation Fund (KP7202104), National Key Research and Development Program of China (2022YFC2505204), and Medical Innovation Research Special Project of Shanghai 2022 Annual Science and Technology Innovation Action Plan (22Y11904500).

## CONFLICT OF INTEREST STATEMENT

The authors declare no conflict of interest.

## References

[1] NCD Risk Factor Collaboration (NCD‐RisC) . Worldwide trends in diabetes since 1980: a pooled analysis of 751 population‐based studies with 4.4 million participants. Lancet. 2016;387(10027):1513‐1530. 27061677 10.1016/S0140-6736(16)00618-8 PMC5081106

[2] Cersosimo E , Solis‐Herrera C , Trautmann ME , Malloy J , Triplitt CL . Assessment of pancreatic β‐cell function: review of methods and clinical applications. Curr Diabetes Rev. 2014;10(1):2‐42. 24524730 10.2174/1573399810666140214093600 PMC3982570

[3] Maggio CA , Pi‐Sunyer FX . Obesity and type 2 diabetes. Endocrinol Metab Clin North Am. 2003;32(4):805‐822. viii. 14711063 10.1016/s0889-8529(03)00071-9

[4] Courcoulas AP , Gallagher JW , Neiberg RH , et al. Bariatric surgery vs lifestyle intervention for diabetes treatment: 5‐year outcomes from a randomized trial. J Clin Endocrinol Metab. 2020;105(3):866‐876. 31917447 10.1210/clinem/dgaa006 PMC7032894

[5] Schauer PR , Bhatt DL , Kirwan JP , et al. STAMPEDE investigators. Bariatric surgery versus intensive medical therapy for diabetes – 5‐year outcomes. N Engl J Med. 2017;376(7):641‐651. 28199805 10.1056/NEJMoa1600869 PMC5451258

[6] Mingrone G , Panunzi S , De Gaetano A , et al. Metabolic surgery versus conventional medical therapy in patients with type 2 diabetes: 10‐year follow‐up of an open‐label, single‐Centre, randomised controlled trial. Lancet. 2021;397(10271):293‐304. 33485454 10.1016/S0140-6736(20)32649-0

[7] Welbourn R , Hollyman M , Kinsman R , et al. Bariatric‐metabolic surgery utilisation in patients with and without diabetes: data from the IFSO global registry 2015‐2018. Obes Surg. 2021;31(6):2391‐2400. 33638756 10.1007/s11695-021-05280-6 PMC8113173

[8] Nguyen NT , Varela JE . Bariatric surgery for obesity and metabolic disorders: state of the art. Nat Rev Gastroenterol Hepatol. 2017;14(3):160‐169. 27899816 10.1038/nrgastro.2016.170

[9] Nannipieri M , Baldi S , Mari A , et al. Roux‐en‐Y gastric bypass and sleeve gastrectomy: mechanisms of diabetes remission and role of gut hormones. J Clin Endocrinol Metab. 2013;98(11):4391‐4399. 24057293 10.1210/jc.2013-2538

[10] Méndez‐Giménez L , Becerril S , Camões SP , et al. Role of aquaporin‐7 in ghrelin‐ and GLP‐1‐induced improvement of pancreatic β‐cell function after sleeve gastrectomy in obese rats. Int J Obes (Lond). 2017;41(9):1394‐1402. 28584298 10.1038/ijo.2017.135

[11] Bancalero‐delosReyes J , Camacho‐Ramírez A , Fernández‐Vivero J , et al. Glucagon‐producing cell expansion in Wistar rats. Changes to islet architecture after sleeve gastrectomy. Obes Surg. 2021;31(5):2241‐2249. 33619692 10.1007/s11695-021-05264-6 PMC8041669

[12] Otero A , Becerril S , Martín M , et al. Effect of guanylin peptides on pancreas steatosis and function in experimental diet‐induced obesity and after bariatric surgery. Front Endocrinol (Lausanne). 2023;14:1185456. 37274331 10.3389/fendo.2023.1185456 PMC10233012

[13] Grün D , Lyubimova A , Kester L , et al. Single‐cell messenger RNA sequencing reveals rare intestinal cell types. Nature. 2015;525(7568):251‐255. 26287467 10.1038/nature14966

[14] Shalek AK , Satija R , Adiconis X , et al. Single‐cell transcriptomics reveals bimodality in expression and splicing in immune cells. Nature. 2013;498(7453):236‐240. 23685454 10.1038/nature12172 PMC3683364

[15] Raza H , John A , Howarth FC . Increased metabolic stress in Zucker diabetic fatty rat kidney and pancreas. Cell Physiol Biochem. 2013;32(6):1610‐1620. 24335379 10.1159/000356597

[16] Liu P , Widjaja J , Dolo PR , et al. Comparing the anti‐diabetic effect of sleeve gastrectomy with transit bipartition against sleeve gastrectomy and roux‐en‐Y gastric bypass using a diabetic rodent model. Obes Surg. 2021;31(5):2203‐2210. 33507518 10.1007/s11695-021-05256-6

[17] Migliorini A , Bader E , Lickert H . Islet cell plasticity and regeneration. Mol Metab. 2014;3(3):268‐274. 24749056 10.1016/j.molmet.2014.01.010 PMC3986629

[18] Gao T , McKenna B , Li C , et al. Pdx1 maintains β cell identity and function by repressing an α cell program. Cell Metab. 2014;19(2):259‐271. 24506867 10.1016/j.cmet.2013.12.002 PMC3950964

[19] Kaneto H , Miyatsuka T , Kawamori D , et al. PDX‐1 and MafA play a crucial role in pancreatic beta‐cell differentiation and maintenance of mature beta‐cell function. Endocr J. 2008;55(2):235‐252. 17938503 10.1507/endocrj.k07e-041

[20] Aigha II , Abdelalim EM . NKX6.1 transcription factor: a crucial regulator of pancreatic β cell development, identity, and proliferation. Stem Cell Res Ther. 2020;11(1):459. 33121533 10.1186/s13287-020-01977-0 PMC7597038

[21] Memon B , Younis I , Abubaker F , Abdelalim EM . PDX1‐/NKX6.1+ progenitors derived from human pluripotent stem cells as a novel source of insulin‐secreting cells. Diabetes Metab Res Rev. 2021;37(5):e3400. 32857429 10.1002/dmrr.3400

[22] Seyfried F , Miras AD , Rotzinger L , et al. Gastric bypass‐related effects on glucose control, β cell function and morphology in the obese Zucker rat. Obes Surg. 2016;26(6):1228‐1236. 26377340 10.1007/s11695-015-1882-5

[23] Peterson QP , Veres A , Chen L , et al. A method for the generation of human stem cell‐derived alpha cells. Nat Commun. 2020;11(1):2241. 32382023 10.1038/s41467-020-16049-3 PMC7205884

[24] Thorel F , Népote V , Avril I , et al. Conversion of adult pancreatic alpha‐cells to beta‐cells after extreme beta‐cell loss. Nature. 2010;464(7292):1149‐1154. 20364121 10.1038/nature08894 PMC2877635

[25] Collombat P , Xu X , Ravassard P , et al. The ectopic expression of Pax4 in the mouse pancreas converts progenitor cells into alpha and subsequently beta cells. Cell. 2009;138(3):449‐462. 19665969 10.1016/j.cell.2009.05.035 PMC2792203

[26] Shiota M , Printz RL . Diabetes in Zucker diabetic fatty rat. Methods Mol Biol. 2012;933:103‐123. 22893404 10.1007/978-1-62703-068-7_8

[27] Perez‐Frances M , van Gurp L , Abate MV , et al. Pancreatic Ppy‐expressing γ‐cells display mixed phenotypic traits and the adaptive plasticity to engage insulin production. Nat Commun. 2021;12(1):4458. 34294685 10.1038/s41467-021-24788-0 PMC8298494

